# Phase I/II study of oxaliplatin with oral S-1 as first-line therapy for patients with metastatic colorectal cancer

**DOI:** 10.1038/sj.bjc.6604271

**Published:** 2008-03-04

**Authors:** Y Yamada, M Tahara, T Miya, T Satoh, K Shirao, Y Shimada, A Ohtsu, Y Sasaki, Y Tanigawara

**Affiliations:** 1 Gastrointestinal Oncology Division, National Cancer Center Hospital, Tokyo, Japan; 2 Gastrointestinal Oncology and Endoscopy Division, National Cancer Center Hospital East, Chiba, Japan; 3 Department of Medical Oncology, Saitama International Medical Center-Comprehensive Cancer Center, Saitama Medical University, Saitama, Japan; 4 Department of Medical Oncology, Kinki University School of Medicine, Osaka, Japan; 5 Department of Pharmacy, Keio University, Tokyo, Japan

**Keywords:** colorectal cancer, oxaliplatin, S-1, SOX, phase I/II

## Abstract

Two phase II studies of S-1 monotherapy have shown promising response rates (RR) of 35–40% with good tolerability in patients with untreated metastatic colorectal cancer. To investigate the usefulness of S-1 plus oxaliplatin (SOX) as an alternative to infusional 5-fluorouracil/leucovorin plus oxaliplatin, the recommended dose (RD) of SOX was determined, and its safety and preliminary efficacy were evaluated in a phase I/II study. Oxaliplatin was administered at a dose of 100 mg m^−2^ (level 1) or 130 mg m^−2^ (level 2) on day 1, and S-1 (80–120) was given twice daily for 2 weeks followed by a 1-week rest. This schedule was repeated every 3 weeks. Level 2 was determined to be the RD. For the 28 patients who received the RD, the median treatment course was 6.5 cycles (2–14), RR of 50% (1 CR and 13 PR: 95% CI 31–69%), with a median progression-free survival of 196 days. Survival rate (1 year) was 79%. Peripheral neuropathy was observed in all patients but with no functional disorders. Major grade 3 or 4 adverse reactions at the RD were neutropaenia (14%), thrombocytopaenia (28%), and diarrhoea (3%). SOX regimen is effective and easily manageable without central vein access.

Oral fluoropyrimidine derivatives have been developed to circumvent the problems associated with continuous infusion of 5-fluorouracil (5-FU). S-1 is an effective derivative that combines tegafur with two modulators of 5-FU metabolism, 5-chloro-2,4-dihydroxypyridine (CDHP), a reversible inhibitor of dihydropyrimidine dehydrogenase (DPD), and potassium oxonate in a molar ratio of 1 : 0.4 : 1 (Kato *et al*, 2001). Tegafur, an oral prodrug of 5-FU, is gradually converted to 5-FU and rapidly metabolised by DPD in the liver. The maximum concentration (*C*_max_) and area under the concentration–time curve (AUC) of 5-FU in plasma during S-1 treatment have been found to be higher than the steady-state concentration and AUC of 5-FU in plasma during protracted intravenous infusion of 5-FU at a dose of 250 mg m^−2^ day^−1^ (Yamada *et al*, 2003).

Potassium oxonate is an orotate phosphoribosyl transferase inhibitor that is distributed primarily to the gastrointestinal tract. This component of S-1 decreases incorporation of 5-fluorouridine triphosphate into RNA in the gastrointestinal mucosa and reduces the incidence of diarrhoea. F-*β*-alanine (FBAL) is the main metabolite of 5-FU. F-*β*-alanine and fluorocitrate are thought to cause the neurotoxic and cardiotoxic effects of 5-FU by inhibiting the tricarboxylic acid cycle (Okeda *et al*, 1990; Robben *et al*, 1993; Diasio 1998). The CDHP component of S-1 inhibits DPD, the rate-limiting enzyme in the catabolic pathway of 5-FU. Consequently, the plasma FBAL concentration after oral administration of S-1 is significantly lower than that after continuous infusion of 5-FU (Yamada *et al*, 2003). Therefore, S-1 may decrease the incidence of neurotoxicity and cardiotoxicity. The response rate of S-1 monotherapy has been found to be 35–40% for patients with metastatic colorectal cancer (Ohtsu *et al*, 2000; Shirao *et al*, 2004), with grade 3 or 4 neutropaenia observed in 5–13%, thrombocytopaenia in 0–8%, diarrhoea in 2–3%, and grade 1 hand–foot syndrome (HFS) in 5%.

Oxaliplatin is a third-generation platinum compound with less toxicity and improved convenience. The regimen of infusional 5-FU and leucovorin (LV) with oxaliplatin is the standard for first- and second-line chemotherapy in patients with metastatic colorectal cancer (de Gramont *et al*, 2000; Rothenberg *et al*, 2003; Goldberg *et al*, 2004). However, infusional 5-FU with LV has the disadvantages of increased inconvenience, cost, and morbidity related to the use of a portable infusion pump and a central venous catheter. Therefore, oral fluoropyrimidine monotherapy has been commonly used in Japan.

The primary objectives of this phase I/II study were to determine the maximum tolerated dose (MTD) of S-1 plus oxaliplatin (SOX). In the phase II study, the toxicity and antitumour activity of SOX were evaluated at the recommended dose (RD).

## MATERIALS AND METHODS

### Patient selection

Patients with histologically confirmed colorectal cancer who had measurable metastatic disease were eligible for the study. Patients with prior chemotherapy and radiotherapy for metastatic disease were not permitted. Patients who had received adjuvant oral fluorouracil-based therapy other than S-1 were eligible if they had remained free of disease for at least 6 months after the completion of such therapy. Other eligibility criteria included an age between 20 and 74 years; an Eastern Cooperative Oncology Group (ECOG) performance status of 0 or 1; adequate baseline bone marrow function (white blood cell count more than the lower limit of normal at each hospital and less than 12 000 *μ*l^−1^, neutrophil count more than 2000 *μ*l^−1^, and a platelet count more than 100 000 *μ*l^−1^), hepatic function (serum total bilirubin (T.Bil) level 1.5 times the upper limit of normal or less, and serum aspartate aminotransferase (AST) and alanine aminotransferase (ALT) 2.5 times the upper limit of normal or less), and renal function (serum creatinine level the upper limit of normal or less); and a life expectancy of at least 90 days. All patients gave written informed consent. Patients were excluded if they had symptomatic brain metastasis, pre-existing watery diarrhoea, or concomitant non-malignant disease, such as cardiac, pulmonary, renal, or hepatic disease, or uncontrolled infection. This study was approved by the institutional review board of each centre. Before enrolment, all patients underwent a physical examination (including documentation of measurable disease), a complete blood cell count (CBC) with differential count, serum chemical analysis, electrocardiography, and computed tomographic (CT) scanning or magnetic resonance imaging (MRI).

### Toxicity and response criteria

Toxicity was assessed according to the Common Terminology Criteria for Adverse Events, Version 3.0 (CTCAE v3.0) (Therasse *et al*, 2000). Neurotoxicity was assessed according to the following specific neurotoxicity grading scale: grade 1, dysesthesia or paresthesia that completely regressed within 6 days; grade 2, dysesthesia or paresthesia persisting for 7 days or longer; and grade 3, dysesthesia or paresthesia causing functional impairment. During the study, all patients were evaluated weekly for signs and symptoms of toxicity. Complete blood cell counts, including differential count, liver function tests, measurement of urea nitrogen, creatinine, and electrolyte levels, and urinalysis were performed weekly. The response of measurable and assessable disease sites was evaluated according to Response Evaluation Criteria in Solid Tumors (RECIST) (Shimoyama, 1999). Tumour dimensions were assessed by CT scanning or MRI every month to confirm response, and after RECIST efficacy was confirmed, every 2 months subsequently.

### Treatment plan

Oxaliplatin was administered as a 2-h infusion every 3 weeks. The duration of the infusion could be extended to 6 h in patients who had pharyngolaryngeal dysesthesia during infusion. S-1 was available in capsule forms containing 20 or 25 mg of tegafur. Patients received S-1 orally twice daily from the evening of day 1 to the morning of day 15 at a dose of 40 mg (BSA<1.25 m^2^), 50 mg (⩾1.25–<1.50 m^2^), or 60 mg (⩾1.50 m^2^) followed by a 7-day rest period in the 3-weekly schedule. All patients received premedication with a 5-hydroxytryptamine-3-receptor antagonist with or without dexamethasone, given as a 30 min drip infusion before chemotherapy. Treatment was routinely given on an outpatient basis. Subsequent treatment was withheld until the neutrophil and platelet counts were greater than 1500 and 75 000 *μ*l^−1^, respectively, AST or ALT less than 150 IU l^−1^, T.Bil less than 1.5 times the upper limit of normal, creatinine less than the upper limit of normal, and diarrhoea, stomatitis, and HFS had resolved to grade 0 or 1. Treatment was repeated until the onset of disease progression or severe toxicity. When the administration of oxaliplatin was discontinued due to oxaliplatin-induced neuropathy, S-1 was also discontinued.

### Dose-escalation schedule

The dose of S-1 was fixed and oxaliplatin was examined at doses of 100 mg m^−2^ (level 1) and 130 mg m^−2^ (level 2). A minimum of three patients were studied per dose level. Dose-limiting toxicity (DLT) was defined as any of the following findings during cycle 1: (i) a neutrophil count of less than 500 *μ*l^−1^ for more than 4 days, (ii) a platelet count of less than 50 000 *μ*l^−1^, (iii) diarrhoea of grade 3 or more that occurred despite adequate supportive therapy, (iv) grade 3 or 4 non-haematologic toxicity, excluding nausea, vomiting, anorexia, and electrolyte imbalance, or (v) a treatment delay longer than 1 week due to drug-related toxicity in the phase I portion. If DLT occurred in one of the first three patients assigned to a given dose level, three additional patients were assigned to the same dose level. The MTD was defined as the dose that induced DLT during cycle 1 in 50% or more of the subjects. The RD was defined as one dose level below the MTD. If the MTD was not achieved, even at level 2, it was regarded as the RD.

The dose was modified for each patient based on haematologic or non-haematologic toxicity. If DLT occurred, the dose of oxaliplatin in the subsequent course was reduced to 75% of the initial dose and that of S-1 was reduced by one dose level: from 80 to 50, 100 to 80, and 120 to 100. S-1 intake was interrupted mid-cycle if there was a neutrophil count less than 1000 *μ*l^−1^, a platelet count less than 75 000 *μ*l^−1^, diarrhoea, stomatitis, or HFS occurred at grade 1 or more, AST or ALT more than 150 IU l^−1^, T.Bil more than 1.5 times the upper limit of normal, or creatinine more than the upper limit of normal. The treatment in the subsequent cycle could be resumed if these adverse events resolved within 3 weeks after the last S-1 treatment. If peripheral neuropathy persisted between courses, the next treatment cycle was started at 75% of the previous dose of oxaliplatin. In a case with pharyngolaryngeal dysesthesia, the duration of the oxaliplatin infusion was prolonged from 2 to 6 h. Recombinant granulocyte colony-stimulating factor was subcutaneously injected if patients had grade 4 neutropaenia or grade 3 febrile neutropaenia, but prophylactic use was not allowed.

### Statistical analysis

The sample size was calculated to be at least 28 patients on the assumption of the null hypothesis of overall response rate of ⩽30% *vs* the alternative hypothesis of overall response rate of >60%, power 80%, and *α* 2.5% (one-sided). The efficacy was analysed by the full analysis set. The primary end point was overall response rate as determined by an External Review Board. The 95% CI for response rate was calculated. Twenty-eight evaluable patients were required. Progression-free survival (PFS) and overall survival were analysed by the Kaplan–Meier method. Safety was analysed in all patients who received at least one dose of study medications. Clinical cutoff date for the study analysis was 31 May 2007.

## RESULTS

### Patient characteristics

A total of 32 patients, 23 men and 9 women, were recruited into this study between March 2005 and June 2006. The median age was 57 years. Four patients had received adjuvant oral fluorouracil-based therapy. Out of 32 patients, 31 received at least one cycle of the study treatment. The demographic data, sites of metastatic tumour, and prior adjuvant therapies are summarised in Table 1. Among the nine patients entered into the phase I study, six patients were treated at the RD. Twenty-three patients entered into the phase II study. However, one patient was excluded from the analysis of efficacy due to symptoms of brain metastasis suspected to have existed before enrolment. All 32 patients were evaluated for toxicity and 28 patients for efficacy.

### Dose-escalation findings

The first three patients were enrolled at dose level 1 (oxaliplatin 100 mg m^−2^, S-1 80–120 mg day^−1^). No DLTs were observed, and six patients were enrolled at dose level 2 (oxaliplatin 130 mg m^−2^, S-1 80–120 mg day^−1^). At level 1, one patient had grade 3 thrombocytopaenia. At level 2, one patient had grade 3 neutropaenia and one patient had grade 4 thrombocytopaenia. The RD was determined to be 130 mg m^−2^ of oxaliplatin in combination with the Japanese standard daily dose of S-1.

### Safety assessment

After identification of tolerability at level 2 (130 mg m^−2^) of oxaliplatin, 29 other patients received the RD at 130 mg m^−2^, including the phase I part patients, to further evaluate the tolerability and toxicity of the study regimen. The median number of administered cycles was 6.5 (range: 2–14), and the total number of cycles for the 29 patients was 180. Oxaliplatin could be administered at the RD without dose reduction in 57% of 28 patients. At the RD, grade 3 neutropaenia was observed in four patients (14%), and grade 3 and 4 thrombocytopaenia in seven patients (24%) and one patient (3%), respectively. The median relative dose intensity was 82.8% for oxaliplatin and 74.6% for S-1 at level 2. The causes of treatment discontinuation at the RD were PD in 13 patients (36%), delayed recovery from toxicity such as neutropaenia, thrombocytopaenia, and slight hyperbilirubinaemia in 8 patients, discretion of the investigator in 2 patients, allergic reaction in 1 patient, and symptomatic deterioration in 1 patient. The treatment was discontinued due to prolonged thrombocytopaenia in eight patients after a median of seven cycles (range: 3–8). No treatment-related death was observed.

Sensory neuropathy occurred in all patients. However, no functional impairment was observed in this study. The most common non-haematologic toxicities were anorexia, nausea, and diarrhoea. One patient had grade 3 diarrhoea at the RD. Another mild adverse event related to treatment was injection site reactions (45%). One patient had severe allergic reactions such as skin rash and fever, which are typical platinum-related reactions during the sixth cycle (Table 2).

### Response to therapy

**Figure 1:**
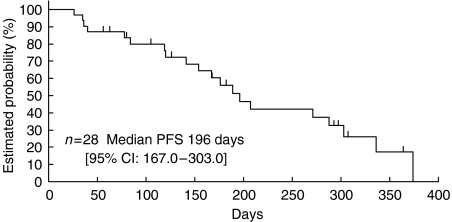
Progression-free survival.

**Figure 2:**
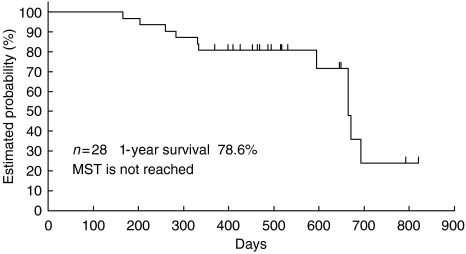
Overall survival.

The objective tumour response was determined by the External Review Board. One of the 28 patients given the RD at level 2 had CR and 13 patients had PR, yielding a response rate of 50% (95% CI: 30.6–69.4%). In the 28 patients studied, the median PFS was 196 days (95% CI: 167–303). The median overall survival time was not reached when 1 year passed since the last patient enrolment, namely 18 patients were alive and 10 patients were dead, and the 1-year survival rate was 78.6% by the Kaplan–Meier method (Table 3) (Figures 1 and 2 ).

## DISCUSSION

Our results suggest that SOX regimen is safe and effective as a first-line treatment for metastatic colorectal cancer. The RD was determined to be 130 mg m^−2^ of oxaliplatin on day 1 with 40–60 mg of S-1 twice daily from the evening of day 1 to the morning of day 15, followed by a 7-day rest period in a 3-weekly schedule. This result indicates that both oxaliplatin and S-1 could be administered at doses similar to those recommended for monotherapy for each drug. SOX regimen has demonstrated promising efficacy with a response rate of 50%, median PFS of 196 days, and a 1-year survival rate of 78.6%. Efficacy of this combination is superior to that reported for monotherapy by each drug (Diaz-Rubio *et al*, 1998; Ohtsu *et al*, 2000; Shirao *et al*, 2004; Boku *et al*, 2007). No DLTs were observed during the first cycle at levels 1 and 2. At the RD (level 2), the toxicity profile was acceptable. The frequent non-haematologic toxicities were anorexia, nausea, and diarrhoea. Most cases of gastrointestinal toxicity were grade 1 or 2, and good oral intake was maintained. There was no grade 3 neurotoxicity observed. Although the incidence of grade 3 or 4 thrombocytopaenia seems to be higher with SOX compared with the reported result of FOLFOX4 (Diaz-Rubio *et al*, 1998; Shirao *et al*, 2004), it was well managed by adequate dose modification of oxaliplatin and S-1 in subsequent cycles (Goldberg *et al*, 2004). Since the severity of thrombocytopaenia is dependent on the dose of oxaliplatin, FOLFOX7 with oxaliplatin at a dose of 130 mg m^−2^ caused 9% of grade 3 thrombocytopaenia (Maindrault-Gœbel *et al*, 2001; Tournigand *et al*, 2006).

The median time to first dose reduction was five cycles (range: 2–7) due to any reason in 16 of the 28 patients at the RD, and 4.5 cycles (range: 3–5) due to grade 3 or 4 thrombocytopaenia in 6 of the 28 patients. Therapy was delayed in 22 of the 28 patients and 40 of 209 cycles, commonly due to neutropaenia, thrombocytopaenia, and sensory peripheral neuropathy. SOX requires only one clinic visit per 3-week cycle for a 2-h infusion of oxaliplatin. This convenience constitutes a marked advantage over regimens combining infused 5-FU/LV by ambulatory pump and oxaliplatin in terms of the impact on the daily lives of patients. In addition, very busy hospitals may have logistic issues providing pumps to all patients; therefore, oral S-1 offers an advantage over infusional 5-FU in respects of convenience and practicability.

The combination regimens of other oral fluoropyrimidine, capecitabine, and oxaliplatin have been reported in other phase II and III studies. Cassidy *et al* (2004) reported the results of a phase II study of oxaliplatin plus capecitabine (XELOX) as a first-line therapy in patients with colorectal cancer (Díaz-Rubio *et al*, 2002). Oxaliplatin (130 mg m^−2^) was administered on day 1 and capecitabine (2000 mg m^−2^ day^−1^) for 14 days with a 1-week rest, every 3 weeks. The response rate, median TTP, and MST were 55%, 7.7 months, and 19.5 months, respectively. Grade 3 or 4 neutropaenia according to NCI-CTC developed in 7% of patients by XELOX, and grade 3 or 4 diarrhoea developed in 16%.

The efficacy and safety of XELOX were also compared with that of 5-FU/LV plus oxaliplatin regimens (FUOX) in several phase III studies. The efficacy of XELOX was statistically not inferior to that of the FUOX regimen: median TTP 8.9 *vs* 9.5 months (*P*=0.153), and MST 18.1 *vs* 20.8 months (*P*=0.145) (Díaz-Rubio *et al*, 2007). Grade 3 or 4 diarrhoea was observed in 14% with both XELOX and FUOX regimens, respectively, and grade 3 or 4 HFS in 2% with XELOX. The efficacy of XELOX was also statistically not inferior to that of FOLFOX4: median TTP 8.0 *vs* 8.5 months, and MST 18.8 *vs* 17.7 months (Cassidy *et al*, 2007). Grade 3 or 4 diarrhoea was observed in 20% with XELOX and 11% with FOLFOX4, and grade 3 HFS in 6% with XELOX and 1% with FOLFOX4. Other schedules of oxaliplatin and capecitabine (CAPOX: 70 mg m^−2^ oxaliplatin on days 1 and 8 and 2000 mg m^−2^ day^−1^ capecitabine for 14 days with a 1-week rest) were compared with FUFOX. CAPOX was slightly inferior to FUFOX in TTP: median TTP 7.1 *vs* 8.0 months (*P*=0.117), and MST 16.8 *vs* 18.8 months (*P*=0.26) (Porschen *et al*, 2007). Both regimens were generally well tolerated, although grade 2 or 3 HFS occurred more often with CAPOX (10 *vs* 4%) (*P*=0.028). In summary, the results of these phase III studies show that the efficacy of XELOX or CAPOX was not inferior to or was slightly inferior to that of infusional 5-FU/LV plus oxaliplatin regimens. Although HFS is more commonly observed in capecitabine-combined regimens, capecitabine is expected to replace infusional 5-FU/LV.

Our limited experience of SOX regimen suggests that tri-weekly treatment with oxaliplatin and S-1 may be comparable to that of XELOX or CAPOX. The response rate of SOX was 50%, suggesting that it is worth while comparing the efficacy of SOX with that of XELOX in the phase III study. Grade 3 or 4 thrombocytopaenia was observed in 28% of patients, and this incidence seems to be higher than that reported by FOLFOX4 (de Gramont *et al*, 2000). Grade 3 or 4 thrombocytopaenia with oxaliplatin monotherapy was reported in 12% of patients (Boku *et al*, 2007), and that with S-1 monotherapy in 0–8% of patients in previous phase II studies (Ohtsu *et al*, 2000; Shirao *et al*, 2004). The most commonly observed grade 3 or 4 toxicity after SOX therapy was cumulative prolonged thrombocytopaenia in this phase I/II trial, which is a well-known toxicity of oxaliplatin. The protocol therapy was discontinued due to prolonged thrombocytopaenia in seven patients after a median of seven cycles (range: 3–8). In cases where sudden and severe thrombocytopaenia is observed, type II allergic reaction to oxaliplatin should be considered and definitive withdrawal is strongly suggested (Maindrault-Gœbel *et al*, 2001). A phase I study of XELOX with 130 mg m^−2^ of oxaliplatin tri-weekly has also shown a relatively higher incidence of grade 3 thrombocytopaenia in 22% (Díaz-Rubio *et al*, 2002), but only 4% during phase II with weekly assessment of CBC (Cassidy *et al*, 2004). Thrombocytopaenia of SOX should be evaluated in the future phase II or III studies with a larger number of patients.

In conclusion, SOX holds promise of being a safe and effective treatment for metastatic colorectal cancer. Further evaluation is expected to examine whether SOX can be a substitute for FOLFOX.

## Figures and Tables

**Table 1:** Patients characteristics

	**Level 1, *n*=3**	**Level 2, *n*=29**
	**L-OHP (100 mg m^−2^)**	**L-OHP (130 mg m^−2^)**
**Characteristic**	**No. of patients (%)**	**No. of patients (%)**
*Age (years)*		
Median	57	57
Range	47–60	34–71
		
*Sex*		
Male	3 (100)	20 (69)
Female	0	9 (31)
		
*ECOG performance status*		
0	3 (100)	26 (90)
1	0	3 (10)
		
*Primary tumour*		
Colon	2 (67)	18 (62)
Rectum	1 (33)	11 (38)
		
*Metastatic site*		
Liver only	1 (33)	10 (35)
Lung	0	3 (10)
Liver and other lesions	1 (33)	10 (35)
Others	1 (33)	6 (21)
		
*No. of metastatic sites*		
1	1 (33)	15 (52)
⩾2	2 (67)	14 (48)
		
*Previous treatment*		
Resection	2 (67)	25 (86)
Adjuvant 5-FU	0	4 (14)

ECOG=Eastern Cooperative Oncology Group; L-OHP=oxaliplatin.

**Table 2:** Toxicity

	**Level 1, L-OHP (100 mg m^−2^), *n*=3**			**Level 2, L-OHP (130 mg m^−2^), *n*=29**		
	**All (%)**	**G3 (%)**	**G4 (%)**	**All (%)**	**G3 (%)**	**G4 (%)**
*Non-haematologic*						
Nausea	1 (33)	0	0	21 (72)	0	0
Vomiting	0	0	0	7 (24)	0	0
Diarrhoea	1 (33)	0	0	17 (59)	1 (3)	0
Fatigue	1 (33)	0	0	25 (86)	0	0
Anorexia	2 (67)	0	0	26 (90)	0	0
Rush	3 (100)	0	0	13 (45)	0	0
Pigmentation disorder	1 (33)	0	0	22 (76)	0	0
Hand–foot syndrome	0	0	0	0	0	0
Peripheral neuropathy	3 (100)	0	0	29 (100)	0	0
Allergic reaction	0	0	0	0	1 (3)	0
						
*Haematologic*						
Neutropaenia	2 (67)	0	0	18 (62)	4 (14)	0
Leukopaenia	2 (67)	0	0	20 (69)	0	0
Thrombocytopaenia	3 (100)	1 (33)	0	27 (93)	7 (24)	1 (3)
Anaemia	1 (33)	0	0	18 (62)	1 (3)	0

L-OHP=oxaliplatin.

**Table 3:** Response rate

	**No. of patients**	**CR**	**PR**	**SD**	**PD**	**Response rate (%)**
*Level 1*						
L-OHP (100 mg m^−2^)	3	0	2	1	0	67 (CI: 9.4–99.2)
						
*Level 2*						
L-OHP (130 mg m^−2^)	28	1	13	9	5	50 (CI: 30.6–69.4)

CI=confidence interval; CR=complete response; L-OHP=oxaliplatin; PD=progressive disease; PR=partial response; SD=stable disease.
